# ARE THERE BENEFITS IN PERFORMING GASTRO-OMENTOPEXY IN LAPAROSCOPIC VERTICAL GASTRECTOMY?

**DOI:** 10.1590/0102-672020210003e1598

**Published:** 2021-12-17

**Authors:** Maíra Danielle Gomes de SOUZA, Lyz Bezerra SILVA, Álvaro A. B. FERRAZ, Josemberg Marins CAMPOS

**Affiliations:** 1 Postgraduate Program in Surgery, Department of Clinical Medicine, Faculty of Medicine, Center for Medical Sciences, Federal University of Pernambuco, Recife, PE, Brazil.

**Keywords:** Obesity, Bariatric surgery, Postoperative Complications, Weight loss, Quality of life, Obesidade, Cirurgia bariátrica, Complicações pós-operatórias, Perda de peso, Qualidade de vida

## Abstract

**Background:**

Gastro-omentopexy promotes the reconnection of the stomach to the gastroesplenic and gastrocolic ligaments and constitutes an alternative for the prevention of complications in laparoscopic vertical gastrectomy.

**Aim:**

To demonstrate the benefits of the gastro-omentopexy technique in patients undergoing sleeve gastrectomy, with possible reduction in postoperative complications.

**Methods:**

Prospective, non-randomized, case series type study, consisting of a clinical population of 179 patients who underwent the technique in 2018, with follow-up between 6-12 months in the postoperative period.

**Results:**

From the participants 71.5% were women, aged between 30-40 years (36.3%). As for the prevalence of complications in the postoperative period, the low prevalence was evident, with emphasis on readmission (1.1%); reoperation (1.1%); wound infection (1.1%); bleeding hemorrhage (0.5%); and stricture (1.1%). However, temporary symptoms were present such as nausea/vomiting, food intolerance, epigastric pain and feeling of fullness, right after surgery.

**Conclusion:**

The technique promoted a significant improvement in quality of life and control of comorbidities. In addition, it was associated with a low prevalence of stenosis, and with no fistula, making the method safer.

## RESUMO

**Racional:**

A gastro-omentopexia promove a reconexão do estômago aos ligamentos gastroesplênico e gastrocólico e pode constituir alternativa para prevenção de complicações na gastrectomia vertical laparoscópica.

**Objetivo:**

Demonstrar os benefícios da gastro-omentopexia em pacientes submetidos à gastrectomia vertical laparoscópica com redução de complicações.

**Métodos:**

Estudo prospectivo, não randomizado, do tipo série de casos, constituído por população de 179 pacientes que realizaram a técnica, com acompanhamento entre 6-12 meses no pós-operatório.

**Resultados:**

Dos participantes 71,5% eram mulheres, com faixa etária entre 30-40 anos (36,3%). Quanto às complicações no pós-operatório evidenciou-se baixa prevalência delas com destaque para reinternação (1,1%); reoperação (1,1%); infecção de ferida (1,1%); sangramento/hemorragia (0,5%); e estenose gástrica (1,1%). Entretanto, surgiram sintomas temporários como náusea/vômito, intolerância alimentar, dor epigástrica e sensação de plenitude, logo após a operação. Houve melhora nas variáveis de qualidade de vida analisadas.

**Conclusão:**

A técnica promoveu melhora na qualidade de vida e no controle das comorbidades. Além disso, esteve associada à baixa prevalência de estenose, e sem ocorrência de fístula, tornando o método mais seguro.

## INTRODUCTION

Laparoscopic sleeve gastrectomy is a surgical technique for the treatment of obesity based on the construction of a gastric tube from the resection of the fundus, part of the body and the gastric antrum; it does not have anastomoses and is mostly restrictive^15^. It has shown good results, becoming the fastest growing bariatric procedure in the USA, and the second most performed in the world, corresponding to 37% of the total^3^.

Sleeve gastrectomy has advantages such as preservation of the gastroduodenal transit and lower risk of nutritional deficiency; however, some postoperative complications can be evidenced, as its performance promotes the disconnection of the gastric tube from the fundamental ligaments for its fixation, such as the gastrosplenic and gastrocolic. Thus, the remnant stomach becomes more susceptible to a reduction in the diameter of the lumen, causing symptoms such as gastric stasis, gastroesophageal reflux, heartburn and regurgitation^24^. In addition, axial rotation may occur, leading to stomach angulation, triggering an increase in intraluminal pressure and the appearance of stenoses, predisposing to the development of fistulas^1^ ^,^ ^6^ ^,^ ^9^ ^,^ ^10^ ^,^ ^23^.

Considering that the absence of fixation of the stomach may determine such complications, gastro-omentopexy has been recently discussed as one of the probable preventive measures^10^ ^,^ ^23^. This technique promotes the fixation of the gastric remnant to the gastrosplenic and gastrocolic ligaments in order to reduce the mobility of the gastric pouch, preventing axial rotation, gastroesophageal reflux disease, hiatal hernia or intrathoracic migration of the gastric pouch. However, there are still few studies in the scientific literature that prove its effectiveness.

The present research sought to demonstrate benefits of performing gastro-omentopexy during laparoscopic sleeve gastrectomy, with possible reduction of complications and improvement in postoperative quality of life.

## METHOD

All participants were informed about the research and signed the informed consent form. This study was approved by the Research Ethics Committee of the Health Sciences Center of the Federal University of Pernambuco (CEP/CCS/UFPE), in accordance with Resolution nº 466/12, of the National Health Council, with opinion nº 3.328. 904, under CAAE No. 11737319.0.0000.5208.

This research was prospective, non-randomized, case series, consisting of a population of 179 patients who underwent sleeve gastrectomy with the laparoscopic gastro-omentopexy technique. All those submitted to it from January to December 2018 in Recife, PE, Brazil were included. The selection of patients followed traditional criteria, such as body mass index (BMI) above 40 kg/m^2^ or above 35 kg/m^2^ associated with comorbidity and age over 18 years.

### Operative technique

The performance of gastro-omentopexy begins with the release of the entire greater gastric curvature through the section of the gastrocolic ligament (near the pylorus), followed by the gastrosplenic; the gastrophrenic ligament is not released. After these releases, the 32-degree Fouchet probe is introduced into the stomach in order to avoid stenosis and guide the staple diameter. The stomach is divided using a 60 mm laparoscopic stapler, the first stapling starts about 3 cm from the pylorus, followed by more shots in the cephalic direction, up to 1-2 cm from the esophagogastric angle. Once the stapling process is completed, the fixation of the entire staple line in the gastrocolic and gastrosplenic ligaments begins, using barbed wire (Stratafix^®^ Ethicon Inc., Somerville, NJ). The staple line of the first 5 cm of the proximal portion of the gastric tube is invaginated with continuous suture. The invaginating suture aims to adjust the diameter, strengthening the region and thus avoiding complications such as fistulas, more common in the proximal portion. Stitches applied to the ligaments are carefully placed in order to avoid vascular damage to the gastro-omental vessels, which pass through the greater curvature of the stomach.

Patients were contacted by telephone to return to consultation, where a structured questionnaire prepared by the researchers was applied, with the following variables: gender, age group, marital status, pre- and postoperative BMI, degree of satisfaction with the weight loss. Follow-up took place between 6-12 months of the postoperative period. The questions regarding quality of life were based on the BAROS protocol questionnaire - Bariatric Analysis and Reporting Outcome System (self-esteem, physical exercise, work motivation, social relationships and sexual interest)^18^. The presence/absence of comorbidities (diabetes, hypertension, reflux, heartburn/heartburn/burning, regurgitation, nausea/vomiting, pain in the epigastrium or chest, feeling of fullness, cough or dysphagia) and the symptoms of reflux in preoperative and postoperative moments, the presence/absence of postoperative complications (rehospitalization, reoperation, surgical wound infection, bleeding/hemorrhage, stenosis, fistula, thoracic pouch migration, axial rotation, gastric volvulus and leaks in the staple line).

### Statistical analysis

The database was built using Microsoft Excel 2010 and exported to SPSS 13.0 (Statistical Package for the Social Sciences) software for Windows. Data were analyzed through the construction of tables and graphs, with their respective absolute and relative frequencies. The chi-square test used for comparisons between proportion/percentage was applied to verify the existence of a comparison between categorical variables in quality of life (satisfaction with weight, self-esteem, physical exercise, work motivation, social relationships and sexual interest) and comorbidities (diabetes, SAH, heartburn, regurgitation, nausea/vomiting, crushing, pain in the epigastrium/chest, feeling of fullness and coughing). In the analysis of repeated measures (moments: preoperative, postoperative and current), the mixed linear regression model was used, which takes into account the possible correlation between the values ​​of the response variable that constitute repeated measures. All tests were applied with 95% confidence and conclusions were obtained considering a significance level of 5% and 95% strength of truth (p=0.05).

## RESULTS

**FIGURE 1:**
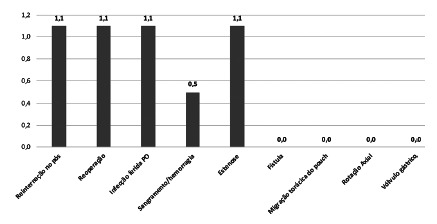
Postoperative complications

A total of 179 patients were evaluated, of which 71.5% were women, with a predominance of the age group between 30-40 years (36.3%). There was a low prevalence of postoperative complications, especially readmission (1.1%); reoperation (1.1%); wound infection (1.1%); bleeding/hemorrhage (0.5%) and stenosis (1.1%). Other complications such as fistula, thoracic bursa migration, axial rotation and gastric volvulus were not present in the study population (Figure 1).

Regarding the prevalence and evolution of comorbidities in the pre- and postoperative periods, there was a reduction in the rates of diabetes (0.6% postoperatively vs. 15.6% preoperatively); hypertension (4.5% postoperatively vs. 36.3% preoperatively) and heartburn (26.3% postoperatively vs. 51.4% preoperatively). However, there was an increase in symptoms such as nausea/vomiting, crushing, pain in the epigastrium/chest and feeling of fullness. There is also a statistically significant difference in almost all the variables analyzed in relation to the moments, except for regurgitation and cough (Table 1).

**TABLE 1:** Prevalence and evolution of comorbidities assessed pre- and postoperatively

	Moments		
Comorbidities	Before	After	p
	n (%)	n (%)	
Diabetes			
Yes	28 (15.6)	1 (0.6)	< 0.001 *
No	151 (84.4)	178 (99.4)	
HAS			
Yes	65 (36.3)	8 (4.5)	< 0.001 *
No	114 (63.7)	170 (95.5)	
Heartburn			
Yes	92 (51.4)	47 (26.3)	< 0.001 *
No	87 (48.6)	132 (73.7)	
Regurgitation			
Yes	21 (11.7)	17 (9.5)	0.493 *
No	158 (88.3)	162 (90.5)	
Nausea/Vomiting			
Yes	5 (2.8)	20 (11.2)	0.002 *
No	174 (97.2)	159 (88.8)	
Dysphagia			
Yes	0 (0.0)	22 (12.3)	< 0.001 *
No	179 (100.0)	157 (87.7)	
Epigastric/chest pain			
Yes	4 (2.2)	16 (8.9)	0.006 *
No	175 (97.8)	163 (91.1)	
Feeling full (Full)			
Ys	3 (1.7)	74 (41.3)	< 0.001 *
No	176 (98.3)	105 (58.7)	
Cough			
Yes	5 (2.8)	6 (3.4)	0.759 *
No	174 (97.2)	173 (96.6)	

*=Qui-Square test

As for the degree of satisfaction in aspects related to quality of life, there was a statistically significant difference (p<0.001) in all variables analyzed in relation to the pre- and postoperative periods. When analyzing the variable weight satisfaction, an inversion is noted: preoperatively 100% of the participants were not very satisfied with their weight, postoperatively 100% became satisfied with their weight loss (Table 2).

**FIGURE 2:**
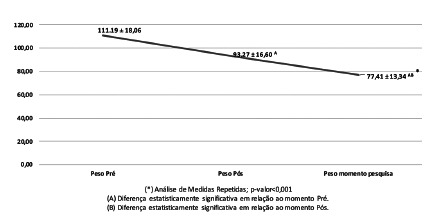
Weight Evolution

In the analysis of the evolution of weight, there was a statistically significant difference after the operation (Figure 2); the mean weight in the preoperative period was 111.19±18.06 kg (weighed at the moment of the survey); at three months after the operation, it was 93.27±16.60 kg and 77.41±13.34 kg in the follow-up between six months and one year after the surgical intervention.

**TABLE 2:** Quality of life assessment regarding the degree of satisfaction in the pre- and postoperative period

	Moments		
Quality of life	Before	After	p
	n (%)	n (%)	
Weight satisfaction			
Little satisfied	179 (100.0)	0 (0.0)	< 0.001 *
Satisfied	0 (0.0)	179 (100.0)	
Self-esteem			
Little satisfied	103 (57.5)	2 (1.1)	< 0.001 *
Satisfied	76 (42.5)	177 (98.9)	
Physical exercise			
Little satisfied	134 (75.7)	47 (26.3)	< 0.001 *
Satisfied	43 (24.3)	132 (73.7)	
Motivation at work			
Little satisfied	80 (44.7)	21 (11.7)	< 0.001 *
Satisfied	99 (55.3)	158 (88.3)	
Social relationships			
Little satisfied	65 (36.3)	23 (12.8)	< 0.001 *
Satisfied	114 (63.7)	156 (87.2)	
Interest in sex			
Little satisfied	61 (34.1)	20 (11.2)	< 0.001 *
Satisfied	118 (65.9)	159 (88.8)	

*=Qui-Square test

## DISCUSSION

Despite the advantages of laparoscopic sleeve gastrectomy, such as sufficient weight loss, absence of anastomoses, accessibility of the stomach and biliary tract by endoscopy, low nutritional deficiency rate and lower risk of dumping, complications such as fistula, stenosis, hemorrhage and reflux gastroesophageal disorders are likely to happen^10^ ^,^ ^13^ ^,^ ^20^. Thus, studies are being carried out to assess the benefits of stomach fixation in sleeve gastrectomy, promoting the reconnection of the gastric remnant to the gastrosplenic and gastrocolic ligaments in order to reduce complications^1^ ^,^ ^9^.

The majority of the population in this study (71.5%) was women, the same fact occurred in the researches carried out by Goulart et al.^14^ in Portugal, where 88.4% of the population was female, and by Pimenta et al.^20^ in Brazil with 61.3%. A study on bariatric surgeries performed by the Brazilian Unified Health System in the period 2010-2016 showed that 85.4% of procedures performed in the country were in women^7^. This fact suggests that women are more careful and concerned about their health when compared to men; in addition, there is aesthetic motivation in society where there are standards of beauty imposed by the media, increasing the demand for and performance of bariatric surgery by this audience^1^ ^,^ ^11^.

The most feared complications in the postoperative period of sleeve gastrectomy are gastric fistula (1-3.9%), hemorrhage (<5%) and stenosis (2-5%). A study by Arslan et al.^4^ showed that gastric symptoms and complications can be minimized with the performance of omentopexy, resulting in a gastric fistula rate of 0.07%, hemorrhage 0.2% and stenosis 0%. Abdallah et al.^1^ carried out a study with 252 patients, divided into control and study groups. In this series, they were able to prove with statistical relevance that the fixation technique reduces the incidence of bleeding from the staple line and axial rotation. A retrospective study with 1200 patients undergoing sleeve gastrectomy and omentopexy found stenosis in 1.33%, bleeding in 0.58%, leakage in the staple line in 0.25% and wound infection in 0.08% as operative complications^5^. Pilone et al.^19^ used two groups, one with omentopexy associated with synthetic sealant, and the other without omentopexy. The overall rate of complications was significantly reduced in the group that underwent omentopexy, suggesting a possible standardization and reproducible approach that can be used to protect the suture for a long time, preventing and reducing complications in sleeve gastrectomy patients. The data published in the literature are similar to this study, where there were no cases of fistula, thoracic bursa migration, axial rotation and gastric volvulus, and low prevalence of some postoperative complications such as readmission (1.1%); reoperation (1.1%); wound infection (1.1%); bleeding/hemorrhage (0.5%) and stenosis (1.1%).

As for the improvement of comorbidities, studies report that bariatric surgery in general can lead to diabetes remission in up to 60% after one year of the surgical procedure; others, specify its remission, using sleeve gastrectomy, between 79-81%^20^ ^,^ ^22^. Thus, the findings in the literature corroborate those of this study, since only one patient (0.6%) maintained diabetes after sleeve gastrectomy with gastro-omentopexy; however, it is essential that these patients maintain long-term follow-up after the surgical procedure. Vargas et al.^25^ demonstrated that 77% of the research participants who presented arterial hypertension in the preoperative period achieved complete resolution of the disease and 38.4% managed to maintain control with a smaller number of antihypertensive medications. In the present study, patients showed clinical improvement in arterial hypertension, which dropped from 36.3% before to 4.5% after surgery.

Regarding heartburn/retrosternal burning, one of the main symptoms of gastroesophageal reflux disease, there was a decline from 51.4% in the preoperative period to 26.3% in the postoperative. Omentopexy, as the literature suggests, sectioning ligaments in sleeve gastrectomy without omentopexy may increase reflux symptoms^17^. However, despite the improvement in heartburn, there was an increase in symptoms such as nausea/vomiting, crushing (related to the consumption of certain foods), pain in the epigastrium/chest and feeling of fullness, especially in the first months of the postoperative period. Dysphagia is a frequent early symptom, due to the phase of food readaptation, with a tendency to decrease in the late period. Some studies have shown that complaints of gastroesophageal reflux can either improve or worsen or even remain after sleeve gastrectomy - in some cases, patients stop using the proton pump inhibitor^2^ ^,^ ^12^ ^,^ ^21^. These findings corroborate the divergences between the authors and point to the need for prospective studies, with a robust sample and more accurate tests, such as esophageal manometry and esophagogastroduodenal seriography, to better assess this outcome.

In the present study, all patients reported significant improvement in aspects related to quality of life, especially in terms of satisfaction with weight loss, in addition to improvement in self-esteem, physical exercise, work motivation, social relationships and sexual interest. The same happens with the data found in the literature, where the scores assessed regarding quality of life showed that patients showed improvement in self-esteem and pleasure in performing routine activities after sleeve gastrectomy^8^ ^,^ ^16^. Pimenta et al.^20^ demonstrated that there was a significant reduction in weight during the three-year follow-up in patients who underwent laparoscopic sleeve gastrectomy, the same happened with the results presented by Goulart et al.^14^, which confirmed the effectiveness of this loss after one year and maintenance of this weight loss 2-3 years after sleeve gastrectomy. In this research, even using a variation of the technique of laparoscopic sleeve gastrectomy with gastro-omentopexy, it also pointed to an evolutionary decrease in weight with follow-up between six months and one year. Importantly, weight loss is similar to sleeve gastrectomy without omentopexy; thus, the choice to use this technique is associated with increased security in the control of postoperative complications in the first weeks or months after the operation.

The study has some limitations, especially regarding the absence of a control group; this is due to the choice of the standard technique of sleeve gastrectomy with omentopexy, performed in all patients. Another limitation is regarding the improvement of reflux, since for a more detailed evaluation of this disease it is necessary to perform more specific tests such as esophageal manometry and esophagogastroduodenal contrast radiological evaluation. In general, gastro-omentopexy in laparoscopic sleeve gastrectomy showed significant results in terms of quality of life and comorbidities. It is a simple and low-cost technique; however, more studies need to be carried out to confirm its effectiveness in terms of reducing complications.

## CONCLUSION

The use of the gastro-omentopexy technique in laparoscopic sleeve gastrectomy was associated with a low prevalence of complications, such as stenosis, and absence of others, as fistula, increasing the safety of patients in the postoperative period.
